# Importance of the Hubbard correction on the thermal conductivity calculation of strongly correlated materials: a case study of ZnO

**DOI:** 10.1038/srep36875

**Published:** 2016-11-10

**Authors:** Anthony Consiglio, Zhiting Tian

**Affiliations:** 1 Department of Mechanical Engineering, Virginia Tech, Blacksburg, Virginia 24061, USA

## Abstract

The wide bandgap semiconductor, ZnO, has gained interest recently as a promising option for use in power electronics such as thermoelectric and piezoelectric generators, as well as optoelectronic devices. Though much work has been done to improve its electronic properties, relatively little is known of its thermal transport properties with large variations in measured thermal conductivity. In this study, we examine the effects of a Hubbard corrected energy functional on the lattice thermal conductivity of wurtzite ZnO calculated using density functional theory and an iterative solution to the Boltzmann transport equation. Showing good agreement with existing experimental measurements, and with a detailed analysis of the mode-dependence and phonon properties, the results from this study highlight the importance of the Hubbard correction in calculations of thermal transport properties of materials with strongly correlated electron systems.

With the increasing demand for inexpensive, yet high preforming materials in nano- and micro-applications, oxides, and specifically ZnO, are becoming promising candidates in fields such as thermoelectrics^1^, optoelectronics^2^, and piezoelectrics^3^. Although much work has already been done to improve the electronic properties of ZnO for applications in piezo- and optoelectronic devices^2^,^4^,^5^,^6^, relatively little is known of its thermal transport properties. Because thermal transport properties play an important role in device modelling and effectiveness, it is important to develop accurate and detailed predictions of thermal conductivity and more fundamentally, phonon propagation mechanics. Unfortunately, reported measurements of the thermal conductivity of ZnO are widely inconsistent, varying from as low as 38 W/mK^7^ to as high as 116 W/mK^8^. A recent calculation^9^ showed minor agreement with lower experimental data from laser flash measurements on unknown sample conditions^7^,^10^,^11^ leading the authors to thus question other higher experimental values obtained by scanning thermal microscopy (SThM)^8^,^12^ along known crystallographic directions of a perfect single crystal. The utilization of a traditional density functional theory (DFT) exchange-correlation functional in their study is, however, questionable. A gap, therefore, still exists in the understanding of ZnO’s thermal transport properties and further work is needed to address this inconsistency.

The electronic structure for materials containing highly correlated electron systems, as is the case with the strong zinc-oxygen interplay in ZnO, is not correctly described by traditional DFT. This problem can be overcome, however, by utilizing the Hubbard U correction (DFT+U), which serves to correct for the unnatural enhancement of the covalent bonds and the misrepresentation of the electronic structure and band gap. Although the band gap is useful in evaluating the correction quantitatively, it is important to note the correct repositioning of electronic bands. In this study, we apply a DFT+U functional to wurtzite ZnO in order to calculate the lattice thermal conductivity and demonstrate that the Hubbard U correction is essential in accurately capturing its thermal transport properties.

Because the inclusion of the Hubbard U term is only a correction to traditional density functional theory, it cannot reproduce any genuine many-body features or alter the physical structure. For this reason, a method that fully considers the on-site correlations, such as dynamical mean-field theory, could theoretically produce even better predictions, yet at a much higher computational cost. This DFT+U method has been applied, however, by Calzolari *et al*. for dielectric properties of ZnO^13^, obtaining good agreement with experimental values, at the same computational cost. The correction, therefore, is a good compromise between accuracy and computational demands, and should be included because the correct description of the electronic band structure and phonon dispersion are important for reliable predictions of the lattice thermal conductivity of strongly correlated materials.

## Methods

DFT calculations were carried out using Quantum ESPRESSO^14^ (QE) and PBE-GGA^15^ ultrasoft pseudopotentials^16^. Initial calculations showed an unnatural enhancement in the covalent nature of the Zn-O bonds, leading to an incorrect description of the electronic structure and severe underestimation of the band gap. This error ultimately stems from the failure of traditional DFT to accurately model strongly correlated systems with pronounced ground state localizations of electrons^17^, and was corrected by implementing a Hubbard U term to partially account for the over-hybridization and localization of electron orbitals. In calculations, Hubbard potentials of U = 12 eV and U = 6.5 eV were applied to the 3d and 2p orbitals of Zn and O, respectively. These values, as reported by Calzolari *et al*.^13^,^18^, successfully correct the wrong energy positions in the band structure, as well as dielectric and vibrational properties. Summarized in Table 1 are the optimized lattice constants and electronic band gap, Δ*Eg*, for PBE and PBE+U with comparison to experiment and a recent study using an LDA^9^ pseudopotential.

**Figure 1:**
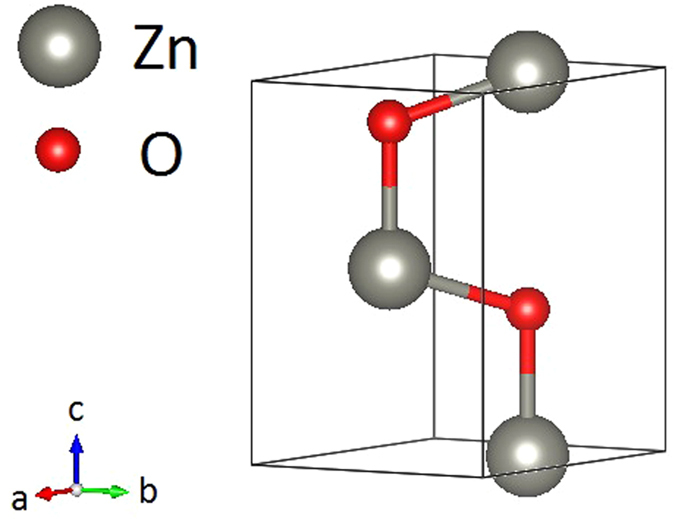
Primitive cell of ZnO with axial directions.

For PBE, density functional perturbation theory (DFPT)^19^,^20^,^21^ was used to calculate the second order interatomic force constants (IFCs), along with the dielectric constants and born effective charges. However, since DFT+U is not feasible under DFPT in QE, a finite difference method was implemented using Phonopy^22^ to obtain the second order IFCs for PBE+U. This was achieved by displacing one Zn and one O atom in the two inequivalent directions (depicted in Fig. 1) in a 4 × 4 × 2 supercell (128 atoms). In this case, to obtain the born effective charges and dielectric constants and apply the non-analytical correction, a series of electric field calculations were performed as described by Calzolari *et al*.^13^.

For calculation of the thirdorder IFCs for both PBE and PBE+U, the thirdorder python code^23^ was used to generate displaced 4 × 4 × 2 supercells. By including the six nearest neighbors and accounting for lattice symmetries, the forces were then calculated by a DFT self-consistent calculation, after which the full anharmonic matrix was reconstructed. Finally, by implementing the software package, ShengBTE^24^, as an iterative solution the Boltzmann transport equation for phonons, the lattice thermal conductivity of bulk crystalline ZnO was calculated on a q-point grid of 30 × 30 × 33 (selected with convergence check). Due to the anisotropy of ZnO, the thermal conductivity was analyzed along both the a- and c-axes as seen in Fig. 1. Additionally, the effect of phonon-isotope scattering was studied due to the many naturally abundant isotopes of ZnO constituent atoms (^64^Zn, 48.6%; ^66^Zn, 27.9%; ^67^Zn, 4.1%; ^68^Zn, 18.8%; ^70^Zn, 0.6%; ^16^O, 99.76%; ^17^O, 0.04%; ^18^O, 0.2%). To include these simulated effects, Tamura’s formula^25^ was implemented, treating mass disorder as perturbation.

## Results and Discussion

Summarized in Table 2 are the calculated dielectric constants and born effective charges for PBE and PBE+U with comparison to experimental data obtained through spectroscopic ellipsometry^26^ and extracted from transverse infrared reflectance spectra^27^. While PBE grossly overestimates the dielectric constants from experimental measurements, PBE+U results match well. The same can be seen for the born effective charges, in which PBE+U predicts more accurate values compared to PBE.

**Figure 2:**
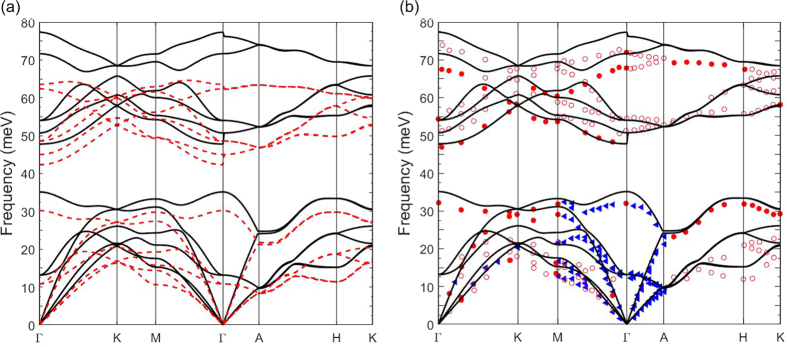
Calculated phonon dispersion of PBE+U (black) and (**a**) PBE (red) and (**b**) experimental inelastic neutron scattering data^28^.

The phonon dispersions for PBE and PBE+U are reported in Fig. 2(a) with comparison of PBE+U to experimentally obtained inelastic neutron scattering values^28^ in Fig. 2(b). While PBE+U shows very good agreement with experimental results, with only the highest bands being slightly overestimated, PBE underestimates the phonon frequencies for nearly every band. Since acoustic modes are the major heat carriers in phonon transport, the slight underestimation of the acoustic bands using PBE in comparison to PBE+U possibly results in notably lower thermal conductivity.

The calculated thermal conductivity using PBE and PBE+U (calculation with exclusion of isotope scattering in parentheses) are summarized in Table 3 along with a recent LDA calculation^9^ and experimental data obtained through SThM^8^,^12^ and laser flash method^7^,^10^,^11^ at 300 K. We found that while PBE, which resulted in thermal conductivities of 39 and 60 W/mK, along the a- and c-axes, respectively, agrees well with LDA^9^, PBE+U resulted in values of 57 and 87 W/mK, and even higher, 63 and 95 W/mK, when not including the effects of isotope scattering. In all cases, however, the calculated thermal conductivity in the c-axis direction was roughly 50% greater than in the a-axis direction. This variation between inequivalent directions can likely explain part of the discrepancy in the measured data.

**Figure 3:**
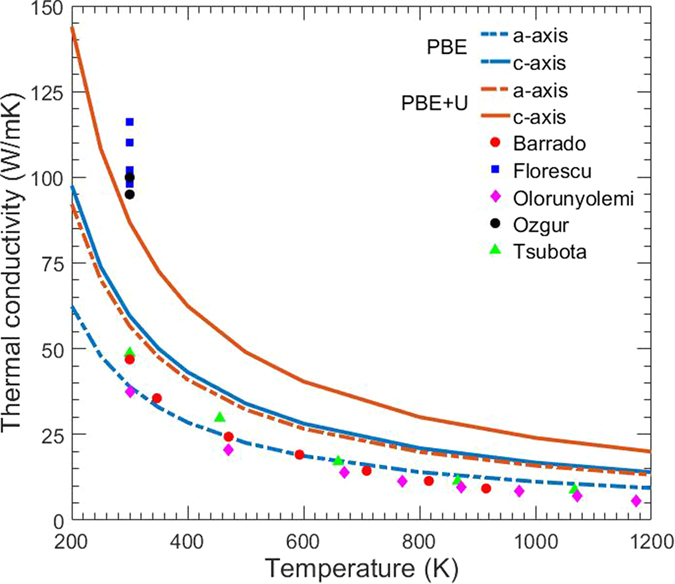
Calculated thermal conductivity for PBE and PBE+U along the a- and c-axis directions with comparison to experimental laser flash^7^,^10^,^11^ and SThM^8^,^12^ data (calculations performed with inclusion of isotope scattering).

Notably, PBE and LDA^9^ agree better with the results from the laser flash method used by Barrado^10^, Oloruneyolemi^7^, and Tsubota^11^. These samples, whose crystal condition and measurement direction were not specified, may have suffered from defects such as grain boundaries, vacancies, and impurities, thus measuring lower values of thermal conductivity. On the other hand, the calculations were performed on perfect single crystals and should in theory give higher values than these measurements. Because of this, the agreements of PBE and LDA^9^ results with laser flash methods are more of a coincidence due to the underestimation in thermal conductivity by PBE originating from the wrong description of the electronic structure in ZnO. PBE+U, however, agrees better with the SThM measurements conducted by Florescu^8^ and Özgür^12^, which measured the thermal conductivity of a single, perfect crystal along the c-axis direction. These trends can also be seen in Fig. 3 below, which shows the calculated anisotropic thermal conductivity for PBE and PBE+U (with inclusion of isotope scattering) along the a- and c-axes with comparison to experimental results^7^,^8^,^10^,^11^,^12^ for a wide range of temperatures.

**Figure 4:**
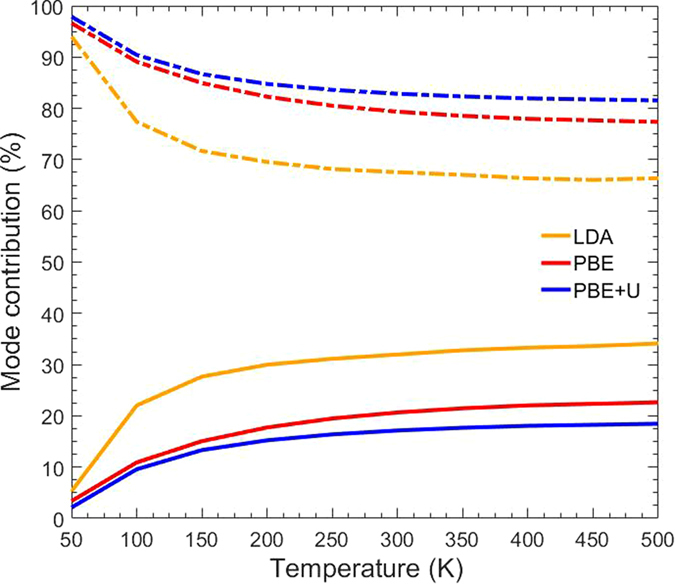
Contribution of optical (solid) and acoustic (dashed) phonon modes to the total average thermal conductivity for LDA^9^, PBE, and PBE+U.

In order to further investigate the discrepancy in thermal conductivity between LDA^9^, PBE, and PBE+U, we analyzed the phonon mode contribution to the total thermal conductivity. Shown in Fig. 4 is the percent contribution of the optical (solid) and acoustic (dashed) modes to the total average thermal conductivity for each of the three pseudopotentials. Although LDA and PBE give similar total thermal conductivities, the detailed contributions are quite different. While PBE and PBE+U show a maximum contribution from optical modes of roughly 20%, the optical modes play a much greater role, ~35%, in the thermal conductivity for LDA. This can possibly explain the discrepancy between LDA and PBE+U since acoustic modes, which have the greatest effect on thermal conductivity, play less of a role in LDA. To understand the difference between PBE and PBE+U, though, other transport properties must be analyzed.

**Figure 5:**
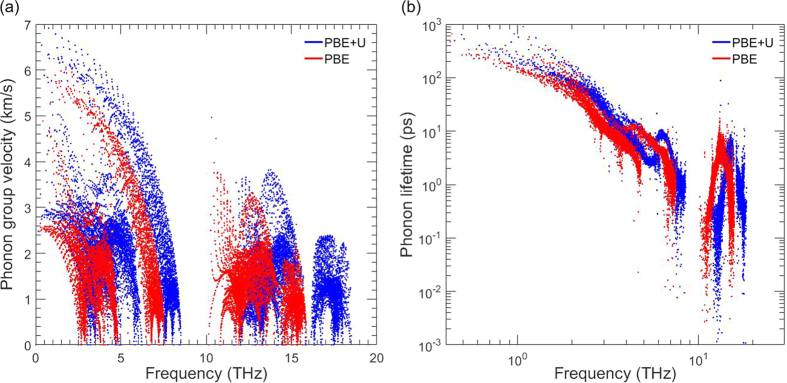
Calculated phonon (**a**) group velocities and (**b**) lifetimes for PBE and PBE+U.

To further investigate the effect of the Hubbard correction, we compared the phonon group velocities and lifetimes between PBE and PBE+U, shown in Fig. 5(a,b), respectively. The phonon group velocities for PBE are lower than for PBE+U when comparing at the same frequencies, as well as when the frequency shift is corrected and the plots are laid over each other. In the case of phonon lifetimes, though, PBE is lower when comparing at the same frequency, but quite comparable when the shift is corrected. In other words, the major discrepancy in thermal conductivity between PBE and PBE+U arises from the difference in phonon group velocities.

**Figure 6:**
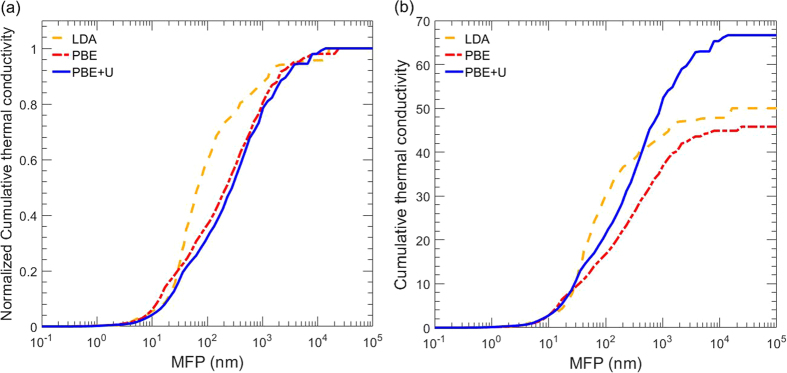
(**a**) Normalized and (**b**) total cumulative thermal conductivity as a function of phonon MFP at 300 K for LDA^9^, PBE, and PBE+U.

Because nanosized defects and grain boundaries greatly affect a material’s thermal properties, it is often useful to analyze the phonon mean free path (MFP) to understand size effects and predict the desired length scales for nanostructuring for reducing thermal conductivity. Shown in Fig. 6(a,b) are the normalized and total cumulative thermal conductivities, respectively, as functions of phonon MFP for LDA^9^, PBE, and PBE+U.

With grain sizes on the order of 100 nm, a reduction in thermal conductivity of 70% and 65% is found for PBE+U and PBE, respectively, while only a 40% reduction is seen for LDA^9^. For all three pseudopotentials, though, mean free paths in the range of 20 nm leads to a reduction in thermal conductivity of 90%. Because of the significant difference in phonon group velocity, it’s a surprising result that the normalized cumulative mean free path accumulations for PBE and PBE+U are so similar.

## Conclusion

In this study we analyzed the effect of Hubbard corrected DFT energy functionals (DFT+U) on the thermal conductivity of ZnO using a PBE pseudopotential, and compared our results to LDA^9^ calculations and experimental laser flash method^7^,^10^,^11^ and SThM^8^,^12^ measurements. We found that both PBE and LDA underestimated the thermal conductivity of ZnO and their agreements with the laser flash method values on unknown sample conditions are more of a coincidence. On the other hand, PBE+U agreed much better with the SThM values, which were conducted on a single, perfect crystal and in a known crystallographic direction. The results of this study ultimately illustrate the importance of the Hubbard correction on accurate prediction of thermal transport properties of highly correlated materials.

## Additional Information

**How to cite this article**: Consiglio, A. and Tian, Z. Importance of the Hubbard-correction on the thermal conductivity calculation of strongly correlated materials: a case study of ZnO. *Sci. Rep*. **6**, 36875; doi: 10.1038/srep36875 (2016).

**Publisher's note:** Springer Nature remains neutral with regard to jurisdictional claims in published maps and institutional affiliations.

## Figures and Tables

**Table 1:** Optimized lattice constants and electronic band gap for pseudopotentials LDA^9^, PBE, and PBE+U.

	a (au)	c/a	Δ*Eg* (*eV*)
LDA^9^	6.02256	1.6166	—
PBE	6.21667	1.615	0.7
PBE+U	6.14459	1.615	3.3
Experiment	6.13972	1.602	3.3^29^, 3.4^30^

**Table 2:** Calculated and experimental^26^,^27^ dielectric constants ( 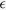 ) and born effective charges (*Z* ^*^) along the a-axis and c-axis (in parentheses).

	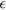	Z^*^
PBE	5.22 (5.33)	2.11 (2.17)
PBE+U	3.08 (3.14)	2.06 (2.12)
Exp	3.70 (3.78)^26^	2.02^27^

**Table 3:** Calculated and experimental thermal conductivity (W/mK) at 300 K along the a- and c-axes for each pseudopotential, including PBE+U without isotope scattering in parentheses.

	a-axis	c-axis
LDA^9^	44	62
PBE	39	60
PBE+U	57 (63)	87 (95)
Barrado^10^	47*	
Olorunyolemi^7^	38*	
Tsubota^11^	49*	
Florescu^8^		98–116
Özgür^12^		95, 100

^*^Direction not specified.
